# Rising methane: is there a methane emergency?

**DOI:** 10.1098/rsta.2021.0334

**Published:** 2022-01-24

**Authors:** E. G. Nisbet, A. E. Jones, J. A. Pyle, U. Skiba

**Affiliations:** ^1^ Department Earth Sciences, Royal Holloway, University of London, Egham TW20 0EX, UK; ^2^ British Antarctic Survey, Madingley Road, Cambridge CB3 0ET, UK; ^3^ Yusuf Hamied Department of Chemistry, Cambridge, CB2 1EW, UK; ^4^ National Centre for Atmospheric Science, UK; ^5^ Centre for Ecology and Hydrology, Penicuik, Midlothian EH26 0QB, UK

## 1. The State of methane

These two volumes of the Royal Society's *Philosophical Transactions* record the ‘state of methane’ in 2021. The atmospheric methane burden rose rapidly in 2020: more rapidly than at any previous time in the observational record. The causes of this rise are complex and not well understood. It is likely much of the growth is driven by increased emissions from biological sources, such as natural wetlands, agriculture and landfills, especially in the Tropics and sub-Tropics. Other processes such as declining methane sinks may also be contributing.

The methane budget is not closed. In the overall estimates by Saunois *et al*. (2020), there are wide uncertainty margins in each sub-category and huge discrepancies between Top-Down and Bottom-Up assessments. Moreover, in seeking to track methane, we are chasing a very fast-changing target—the global methane budget in 2021 is very different from the budget in 2010.

## 2. Is methane measurement adequate?

At present, global monitoring is limited to the NOAA network and a small number of rigorously intercompared allied measurement programs. There are few long-term time series for mole fraction and even fewer high precision multi-sample monitoring stations for*δ*^13^C_CH4_.

To resolve ‘Top-Down’ budgets the requirements are for:

(1) Better long-term observational time series, for both mole fractions and isotopes, including extended isotopic monitoring of both *δ*^13^C_CH4_ and *δ*^2^H_CH4_ to provide data on both methane mole fraction and isotopes at an adequate number of remote monitoring stations measuring background air. There is also a need for closer linkage of on-ground *in situ* measurement, aircraft-based regional measurement, and satellite observation of the total column. Global ‘Top-Down’ methane budgets are determined by Bayesian modelling but presently, Bayesian inversions of global and regional budgets are poorly constrained because the data are so few, especially for isotopes.
  In the Tropics, there are few long-term measurements of background air. For example, one of the few multi-decadal time series from the equatorial zone is from Ascension, the Atlantic equivalent to Mauna Loa in the Pacific. This provides the background against which Amazonian emissions are assessed. Critically needed is secure long-term funding to monitor the tropical atmosphere, measuring atmospheric gases linked to climate change and air pollution, as part of the UN Global Atmospheric Watch.
(2) Better characterization, worldwide, of the isotopic source signatures of major sources, by nature and geographic location. Arctic wetlands have *δ*^13^C_CH4_, around −70‰, while methane from tropical wetlands has *δ*^13^C_CH4_ around −50 to −55‰, and from cows around −55‰. Gasfield *δ*^13^C_CH4_ signatures also vary greatly, so that gas sources can be identified isotopically.
  To improve global and regional modelling and source identification, *δ*^2^H_CH4_ provides powerful information but measurement of *δ*^2^H_CH4_ has barely begun. Although NOAA did for some years maintain a small network of remote stations measuring *δ*^2^H_CH4_, that network had to be closed and currently, only a handful of stations maintain *δ*^2^H_CH4_ time series. Recently, in the MOYA programme, we have developed a rapid *δ*^2^H_CH4_ measurement capacity that is able to carry out large numbers of high precision samples for routine monitoring inexpensively. For the future, *δ*^2^H_CH4_ offers much: a potent tool in constraining global and regional budgets.
(3) Regular methane surveys by low-flying aircraft, to sample and quantify regional emissions, both over wetlands and agricultural areas. The MOYA/ZWAMPS campaigns for fluxes and regional isotopic signatures in Senegal, Uganda and Zambia and Bolivia showed the power of such flights. Regional fluxes can be estimated and regional isotopic signatures can be defined. For example, summer emissions in Zambia and lowland Bolivia have *δ*^13^C_CH4_ around −60‰, while summer emissions in northern Scandinavia have a regional signature of *δ*^13^C_CH4_ −73‰.
(4) Better ‘prior’ estimates about emissions and source signatures, to inform the Bayesian inversion problem. Currently, the United Nations Framework Convention on Climate Change (UNFCCC) gathers detailed, frequently updated, Bottom-Up emissions data from so-called Annex-1 nations, for example, Europe and the US, but the non-Annex 1 nations, including China, India and Brazil, are only required to give out-of-date estimates. In default, most academic studies instead use the European Union's excellent Emissions Database for Global Atmospheric Research ‘*EDGAR*’ as well as UNFCCC data and other information.

## 3. Is the warming feeding the warming?

Is the warming feeding the warming? Will methane emissions rise in response to climate warming? The answer is not known: it depends on what exactly is driving growth. A warmer world is also a wetter world. Wetland emissions grow both with temperature and with wetland area. Thus, a feedback is likely and expected. Tropical and Boreal/Arctic wetland sources will most probably emit more methane.

As the planet warms, becoming wetter and hotter, and the Tropics expand, tropical farming will become more productive. An increase in human agriculture will likely occur with more ruminants, more crops and crop waste fires, more use of fertilizer running off into wetlands, all leading to methane emission.

Fossil fuel methane emissions may also respond to climate warming. Although warmer winters in the North may lead to a decline in the use of gas for heating, warmer Tropical and sub-Tropical summers will occur. Air conditioning is now very widespread across the tropics and sub-tropics and will drive rising demand for electricity, much of it currently gas- or coal-fired with attendant methane emissions.

## 4. Mitigation and removal

Methane's lifetime in the atmosphere is less than a decade: emissions are naturally destroyed in a few years. In general, the best way to reduce methane is to stop emission. Nevertheless, there are also strong benefits from methane removal in particular locations where high methane mixing ratios are persistent. Thus, both mitigating emissions, and also enacting atmospheric methane removal, represent powerful levers to slow global warming.

Gas leaks, coal mine emissions, oilfield vents and flaring; manure emissions from industrial-scale cattle, pig and chicken farms; methane from badly managed landfills, especially in the Tropics: for these, mitigation is easy. Crop waste fires are harder to control, but there are large cross-benefits from controlling major sources of air pollution e.g. in India and Africa.

Most pasture land is marginal land. Ruminants produce food from land unsuited for arable farming. They are essential to sustain food supplies, but also are a significant global source of methane. Without ruminants, food supplies would have to depend on crop land, likely with expansion. Most cattle are Tropical or sub-Tropical (India, Africa, South America) and culturally important in many nations. It is unlikely that any significant reduction in milk demand will occur until human population growth slows, especially in moist tropical Africa, although it may be possible to cut meat over-consumption.

Atmospheric methane removal has had little attention. While concentrations globally are relatively dilute for economic removal, it is likely advantageous to remove methane from locations where air habitually has high methane mole fraction—say 50–100 ppm—such as in cattle barns, around essential sewage or waste facilities, and in some fossil fuel industry locations. ‘Net Zero’ human methane emissions are perhaps unattainable, given our need for food, but removal may help make inroads towards that target.

## 5. Is there a methane emergency?

In British law, an ‘*Emergency*’ is ‘*an event or situation which threatens serious damage to human welfare … , the environment of a place … .or … serious damage to … security*’. Dictionary definitions typically add the words ‘*unexpected*’ and ‘*sudden*’. As the rise in the atmospheric methane burden meets all those criteria, there is indeed a methane emergency.

## 6. Inferences

Have we made progress? Has the work reported in these volumes of *Phil. Trans. R Soc. A* and the parallel discussion meeting made the methane problem more accessible? Yes: the methods of measurement, including isotopic measurement, and regional and global modelling are much improved. Urban and fossil fuel emissions are better constrained. However, the OH problem—is the oxidative (self-cleansing) capacity of the global atmosphere changing or declining?—remains a major gap in understanding.

In particular, we have learned much about tropical emissions, especially in Africa where there has been very little previous study. The implications are that if the methane problem is to be addressed, then the tropical nations are not simply bystanders, as they largely are for CO*_2_*—they need actively to help reduce emissions, for example, by covering landfills and reducing crop waste burning and deforestation by fire. This will need the development in tropical nations of local constituencies of scientific understanding, together with better access to equipment (imports are often blocked by local import controls and customs processes) and to satellite mapping linked to atmospheric transport modelling.

Methane is rising. Though strong positive feedback is likely, with emissions growing as the climate warms, powerful action on anthropogenic emissions can indeed halt that rise and reverse it. Substantial emission reductions can quickly be made: by ending emissions from the coal, gas and oil industries, by cutting emissions from cow and pig manure stores, by removing methane in cattle barns, by cutting leaks from biodigesters, by ending crop waste fires and by covering landfills, not only in developed nations but also in the tropics. Though ‘Net Zero’ methane is unlikely this century, much can be done.

## Data accessibility

This article has no additional data.

